# Reduced Contextual Discrimination following Alcohol Consumption or MDMA Administration in Mice

**DOI:** 10.1371/journal.pone.0142978

**Published:** 2015-11-13

**Authors:** Emily M. Johansson, María S. García-Gutiérrez, María Moscoso-Castro, Jorge Manzanares, Olga Valverde

**Affiliations:** 1 Neurobiology of Behaviour Research Group (GReNeC), Department of Health and Experimental Sciences, Universitat Pompeu Fabra, IMIM, Hospital del Mar Medical Research Institute, Barcelona Biomedical Research Park C/Dr. Aiguader 88, 08003, Barcelona, Spain; 2 Instituto de Neurociencias, Universidad Miguel Hernández-CSIC, Avda. Ramón y Cajal s/n, San Juan de Alicante, Alicante, Spain; 3 Red Temática de Investigación Cooperativa en Salud (RETICS), Red de Trastornos Adictivos, Instituto de Salud Carlos III, Madrid, Spain; Oregon Health and Science University, UNITED STATES

## Abstract

The recreational drugs, alcohol and 3,4-Methylenedioxymethamphetamine (MDMA, “Ecstasy”) have both been shown to cause immune activation *in vivo*, and they are linked to cognitive impairment and anxiety-like behaviors in rodents. The neuronal effects of these drugs in the hippocampal area, an area that has been a focus of studies aiming to explain the mechanisms underlying anxiety related-disorders, remains poorly understood. Therefore we investigated the specific inflammatory impact of alcohol and MDMA on this area of the brain and on a hippocampal-related behavioral task. We centered our study on two inflammatory factors linked to anxiety-related disorders, namely Interleukin-1β (IL-1β) and brain-derived neurotrophic factor (BDNF). We subjected drug-consuming mice to a battery of behavioral tests to evaluate general activity, anxiety-like and depressive-live behaviors. We then introduced them to a contextual fear discrimination task and immune-related effects were examined by immunohistochemical and biochemical studies. Our results suggest that there is a relationship between the induction of immune activated pathways by voluntary alcohol consumption and a high-dose MDMA. Furthermore, the ability of mice to perform a contextual fear discrimination task was impaired by drug consumption and we report long term inflammatory alterations in the hippocampus even several weeks after drug intake. This information will be helpful for discovering new selective drug targets, and to develop treatments and preventive approaches for patients with anxiety-related disorders.

## Introduction

Considerable immune activation is observed in the brain following the use of either alcohol or 3,4-Methylenedioxymethamphetamine (MDMA) [1–5], and the relationship between neuroinflammation and mood disorders is well recognized (for review see [6,7]). Notably, high-risk consumption, or binge drinking, is common among teenagers throughout Europe and the US [8] and furthermore, alcohol is frequently consumed together with other drugs of abuse, particularly psychostimulants like MDMA/“Ecstasy” [9–11]. The effects of both drugs on emotions are well documented (for review see [12–14]). In brief, alcohol-dependent individuals are three times more likely to have an anxiety disorder or depressive symptoms than the general population [15] and a single MDMA dose can provoke panic attacks in individuals with no previous psychiatric history [16]. Anxiety disorders, including panic attacks, are among the 10 leading causes of years lost due to disability [17], and therefore, the understanding of the effect of MDMA and alcohol on anxiety-related behavior is of particular interest.

The proinflammatory cytokine Interleukin-1β (IL-1β) has gained particular attention due to its importance in memory related processes like hippocampal neurogenesis [18] and synaptic plasticity [19]. Furthermore, the release of brain-derived neurotrophic factor (BDNF), which is also involved in adult neurogenesis, neuronal plasticity and survival [20,21], is regulated by IL-1β among others [22]. Indeed, patients with panic disorder, a specific subgroup of anxiety-related disorders, show alterations in both the neurotrophic factor and IL-1β [23,24].

One hypothesis regarding the underlying mechanisms of panic disorders has evolved from the observation of cognitive impairments related to hippocampal functions [25]. This is manifested by these patients’ inability to learn how to distinguish between a safe environment and a dangerous one [25]. Animal studies using a hippocampal-dependent contextual fear discrimination (CFD) task are aimed to evaluate the ability of mice to learn to discriminate between two very similar contexts [25]. Taken together, we were interested in studying the impact of the two drugs (EtOH and MDMA) on the inflammatory environment in the hippocampus and on hippocampal-related behavioral tasks.

For this, we focused on evaluating alterations in the expression of IL-1β and BDNF (both associated with anxiety and neuroinflammation [26–28]) and we used the CFD-task as a behavioral paradigm. We related impaired contextual discrimination to disturbance of hippocampal functions and to the inability of the mice to suppress fear in the presence of safety cues. Previous studies in our laboratory show that both drugs cause oxidative damage to specific proteins related to structural functions and outgrowth in hippocampal areas, and that acute MDMA administration but not EtOH consumption affect the performance of mice in the radial maze and object recognition task [29]. However, the specific inflammatory reactions in the hippocampus and hippocampal related behavioral consequences of drug intake have not been fully addressed to date.

We found altered IL-1β and BDNF expression in the hippocampus as well as behavioral perturbation in mice that had consumed either EtOH or received a high-dose MDMA.

## Material and Methods

### Animals

Male mice (Swiss ICR) were purchased from Harlan (Barcelona), and were matched for age and weight (2–3 months; weight 25–30 g). Seven days before starting the experiments, mice were individually housed, and the 12-h light/dark cycle was reversed (light on 7:00 PM). Mice were kept at constant ambient temperature (21±1°C) and humidity (55±10%) with *ad libitum* access to food and water. Every effort was made to minimize the number of animals used and their suffering. Animal procedures were conducted in accordance with the European Community guidelines (Directive 86/609/EEC) regulating animal research, and were approved by the local Clinical Research Ethics Committee (CEEA-PRBB).

### Experimental procedures

Following drug exposure, mice were injected one a day for three consecutive days with bromo-deoxyuridine (BrdU) (Sigma-Aldrich, Madrid, Spain) (150 mg/kg, 10 ml/kg, dissolved in saline PH≈7.35, i.p.) before being exposed to the drinking in the dark (DID)-procedure. Only animals assigned for euthanasia 72 h after drug consumption were injected with BrdU, while others received saline. The DID-procedure was conducted as previously reported [29–31]. Briefly, 3 h after the lights were turned off, food was removed and the water bottles were replaced with 10-ml graduated cylinders fitted with sipper tubes containing either 20% (v/v) ethanol in water, or water only. Individual intake was recorded after 2 h and food and water bottles were replaced. This procedure was repeated for 3 days and fresh fluids were provided each day. Any tube with a registered drinking volume of more than 2 standard deviations from mean was considered as spill, and the record was removed from the data. On day 4, the period of alcohol consumption was extended by 2 h, and all subjects were injected with a single dose of saline 30 min prior to the first period and immediately before the second 2-h period of drinking, to mimic the situation with MDMA injections the following week. The animals were divided into 3 groups: the Control-group (water and saline injection, n = 12), the EtOH-group (EtOH and saline injection, n = 12), and the MDMA-group (water and MDMA injection, n = 12). In a second round of experiments, animals were assigned to the same 3 groups (n = 5-8/group) and kept for 22 days after drug consumption without behavioral testing before euthanasia.

#### MDMA administration

The dosage (20 mg/kg x 2) of racemic MDMA hydrochloride (Spanish Agency for Medicine, Ministry of Health) was selected in accordance with previous studies from our laboratory [29,31]. On day 4, basal body temperature was measured rectally using an electronic thermo-coupled flexible rectal probe (Panlab, Madrid, Spain) 30 minutes before starting the DID-test. This was followed by a single dose of MDMA (20 mg/kg) or saline (i.p.). After the first recording of liquid intake (2 h), body temperature was measured and animals received a second injection of MDMA (20 mg/kg) or saline. Room temperature was held at 21±1°C, and changes in body temperature with respect to baseline temperature were calculated for each mouse and time point.

### Behavioral responses

Experiments were conducted during the dark cycle by the same experimenter as the one who handled the animals during the day. Each group of animals was subjected to a battery of behavioral tests, in the following order: (1) locomotor activity, (2) anxiety-like behavior in the elevated plus maze (EPM), and (3) despair-like responses in the tail suspension test (TST), leaving at least 1.5 h between each test. Behavioral tests were conducted 72 h after the last MDMA/saline injection, and were followed by euthanasia 12h after the TST (6 animals from each group). Inflammatory responses were evaluated in euthanized animals (see below), while the remaining 6 animals/group continued to the CFD-test. Mice were randomly assigned to either the immunohistochemistry or CFD groups.

All animals were naive to the apparatus when first presented with the tests, and no habituation was performed. The experimenter was blind to the animal’s condition during all tests (n = 12 mice per group or n = 6 for CFD).

#### Actimetry box test

Locomotor activity was measured in actimetry boxes (25x25cm; Panlab s.l.u., Barcelona, Spain). Movements were monitored by a grid of infrared beams and used as an index of locomotor activity (counts). Counts were integrated twice after 10 min each in almost complete darkness (5 lux), and were summed to obtain total horizontal and vertical movements over a period of 20 min. All data were collected using Seda-Com software (Panlab s.l.u.).

#### Elevated plus-maze

This apparatus (Panlab s.l.u.) consisted of a plastic maze with four arms (16 cm wide x 65 cm long) radiating from a neutral central square (5x5 cm). Two opposite arms were enclosed by vertical walls (closed arms) while the other two arms had unprotected edges (open arms). The maze was elevated 30 cm above the ground and placed under indirect light in open (100 lux) and closed (30 lux) arms. The procedure was conducted as previously described [32]. Each mouse was placed in the central square facing one of the open arms and was observed for 5 min. An arm entry was recorded when the mouse moved four paws into the arm. The total number of entries and the cumulative time (in seconds) spent in the open and closed arms were recorded manually.

#### Tail suspension test

Despair like behavior was evaluated using the TST, as previously described [33]. Briefly, mice were individually suspended by the tail from a horizontal ring-star bar (35 cm from the floor) using adhesive tape (2 cm from tip of tail). The cumulative number of seconds spent immobile during a total time of 6 min was recorded.

#### Contextual fear discrimination

The protocol for testing CFD learning was based on previously published protocols [34,35]: two compartments with similar features were used; the experimental chamber (25x25x25cm) was made of black methacrylate walls, a transparent front door and a stainless steel grid floor (Panlab s.l.u.). Context B differed from context A in that the walls were covered using plastic inserts (made from ethylene vinyl acetate) that created a convex wall, and the white noise and lights were turned off. In addition, the chamber door was left partly open and the explicit smell from the plastic inserts was used as an olfactory cue instead of the mild lemon scent used in context A.

Animals were transported in the dark to the experimentation room at least 1 h prior to testing. Each animal was brought to the chamber from the opposite side of the room (divided by a curtain) in its respective cage under a dark blanket. On days 1–3 during context acquisition, the animals were kept in context A, and 3 min into the session they received a 0.7 mA shock during 2 seconds, and were then left in the context for 15 additional seconds after the shock. We measured freezing during 3 minutes before shocking each day throughout the test, from day 1 to day 17. A generalization test was conducted on days 4–5 of testing to verify that the animals displayed extensive generalization between the two contexts. This test consisted of two 3-min sessions where mice were exposed to both contexts A and B in a counter balanced order with no shock in either compartment; these sessions were separated by a 1.5–2 h interval. The CFD test was initiated on day 6, and on days 6–17 the mice were exposed to both contexts each day, with a 1.5-2h interval between each exposure. We randomly selected the first context for each day as follows: BAABABBABAAB, i.e. on days 7, 8, 10, 13, 15 and 16 all animals were exposed first to Chamber A and then to Chamber B, and the reverse on the remaining days. Measuring freezing in both context A (3-min pre-shock, 0.7mA) and context B (3 min, no shock) each day allowed us to evaluate the mice’s capacity to discriminate between the two contexts.

### Tissue processing

Twelve hours after the TST and 4 days after drug consumption (n = 6/group), brains were removed, divided into two hemispheres, frozen rapidly on dry ice, and stored at -20°C. After completion of the CFD trial (22 days after drug consumption) the brains were transferred to ice cold artificial cerebral fluid for dissection of dorsal and ventral hippocampal areas, and then frozen on dry ice. In a second set of experiments, sections of the hippocampus from animals naïve to the behavioral battery were dissected at 22 days after drug consumption, and the frozen sections were later processed for PCR studies, as described below. For immunohistochemistry and staining, 16-μm-thick coronal tissue sections of one hemisphere were cut on a microtome-cryostat (Microm HM500 OM, Walldorf, Germany), thaw-mounted on StarFrost slides (Knittleglass, Braunschweig, Germany), and stored at -20°C until further processing.

#### Immunohistochemistry

The following antibodies were used: rabbit polyclonal anti-ionized calcium binding adaptor molecule 1 (Iba1) (Wako, Neuss, Germany, dilution 1:300) to detect microglia/macrophages; rabbit polyclonal anti-glial fibrillary acidic protein (GFAP) (DakoCytomation, Glostrup, Denmark, dilution 1:800) to detect astrocytes; and rat anti-BrdU (Abcam, Cambridge, UK, dilution 1:300). Immunohistochemistry was performed as previously described [36], with changes for BrdU labeling: the tissue was treated with 4N HCl for 15 min at R.T. after fixation and then neutralized in tap water. Sections were mounted using Mowiol.

#### Gene expression analyses: Real time PCR

Brain sections from mice sacrificed 12h after behavioral studies (4 days after drug consumption) were cut (500 μm) in a cryostat (-10°C) at levels containing the hippocampus according to Paxinos and Franklin [37], mounted on slides and stored at −80°C; sections were dissected according to the method described by Palkovits [38]. For mice sacrificed 22 days after drug consumption and 4h after completion of CFD task (at day 22), we used the hippocampal dissections. Total RNA was isolated from brain tissue micropunches using TRI Reagent^®^ (Ambion, Madrid, Spain), and subsequently retrotranscribed to cDNA. Quantitative analysis of the relative abundance of IL-1β and BDNF gene expression was performed using the Step One Real Time PCR System (Applied Biosystems, Madrid, Spain). All reagents were obtained from Applied Biosystems and used according to the manufacturer’s protocols. We used 18S rRNA as the reference gene, detected using Taqman ribosomal RNA control reagents. Data for each target gene were normalized to the endogenous reference gene, and the fold change in the abundance of target gene mRNA was determined using the 2^(-ΔΔCt)^ method [39].

### Preparation of figures and analysis

To count BrdU-labeled cells, five sections each from the dorsal and ventral parts of the hippocampus, at approximately 100μm intervals, were processed for immunohistochemistry. BrdU-labeled cells in the dentate gyrus (DG, granule cell layer) were counted at x20 under a light microscope (Leica DM6000 microscope, Wetzlar, Germany) by an experimenter blinded to treatment. Cell counts from all sections/animal for both the dorsal and ventral parts of the DG were summed to obtain the “total” cell count per DG. Immunohistochemistry images were digitized using a Photomotrics CoolSNAP HQ^2^ digital camera (Tucson, AZ, USA), Nikon NIS Elements imaging software, and a Nikon eclipse T*i* microscope (Nikon, Tokyo, Japan). The GFAP- and Iba1-stained areas (4 animals/group) were quantified as the cell counts and the fraction of the total area, respectively, above a given intensity threshold using Fiji ImageJ Software (http://fiji.sc/Fiji). Figures were assembled using Adobe Photoshop (Adobe Systems, San Jose, CA); images were optimized using contrast and brightness only.

Liquid intake (ml) was analyzed using two-way ANOVA followed by Bonferroni’s post hoc test for treatment and day, and water and EtOH consumption (g/kg or ml/mean of 3 days) using the Paired t-test. The results of behavioral testing were analyzed using one-way ANOVA followed by Bonferroni’s post hoc test for treatment. The results of the CFD test were analyzed using repeated measures (RM) one- and two-way ANOVA (missing values were treated using regression substitution), followed by Bonferroni’s post hoc test for treatment and day. Immunohistochemistry and biochemistry data were analyzed using one-way ANOVA followed by Tukey’s post hoc test. Differences were considered significant when the probability of error was less than 5%. All statistical analyses were performed using GraphPadPrism 5 (GraphPad Software, San Diego, CA).

## Results

**Fig 1:**
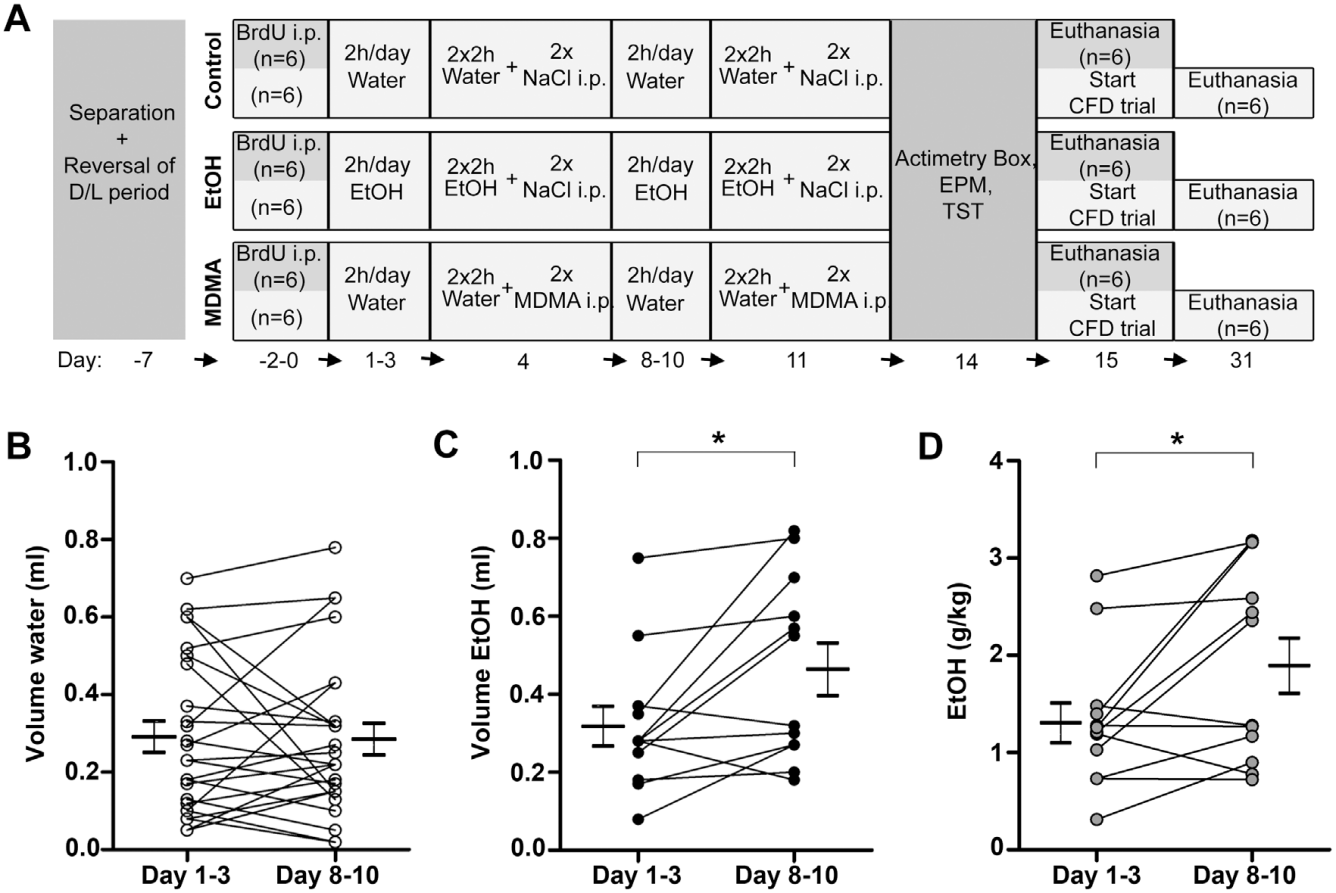
Experimental timeline and drinking behavior. (**A**) Each stage of the experiment is separated by a line and the day(s) of each stage is shown underneath. Mice were deprived of food but had free access to either water or EtOH (20%) for 2h per day or for 4h on days 4 and 11. On day 11, water and EtOH drinkers were injected (i.p.) with MDMA (20 mg/kg x 2) or saline, respectively, with 2h in between. Locomotor activity, anxiety-like and despair-like behaviors were evaluated 72 h and 4 days after the last MDMA or saline injection. The following morning 6 mice/group were euthanized and 6 mice/group continued to the CFD trial. Daily volume (ml) of (**B**) water or (**C**) EtOH intake registered, and (**D**) EtOH consumption, calculated as g/kg, (n = 12–24 mice/group) are shown. Statistically significant differences in EtOH intake between days 1–3 and 8–10 are indicated by brackets and are represented by C *p<0.0499 and D *p = 0.0241); Paired t-test. The mean ± SEM is shown at the sides. CFD, contextual fear discrimination; EPM, elevated plus maze; MDMA, 3,4-Methylenedioxymethamphetamine; TST, tail suspension test.

Drinking behavior was registered 4 days/week and on day 4 two saline injections were given to all mice. On day 11 two high-dose MDMA injections were given to mice drinking water, while mice drinking EtOH were given two saline injections. 72h after drug consumption, we conducted behavioral evaluation of locomotion, anxiety-like and despair-like responses, followed by either a contextual fear conditioning task or euthanasia for immunohistochemical and biochemical evaluation (Fig 1A and S1 Fig). Our results show an increased liking for EtOH throughout the DID-test. Furthermore, the capacity of the mice to perform a contextual fine discrimination task was not only obstructed by a high MDMA dose but also by voluntary alcohol intake during several consecutive days.

### Alcohol and MDMA consumption

In the DID test mice generally drank a lower volume of water (Fig 1B) than EtOH (Fig 1C) (Student's t-test: *p* = 0.0481). EtOH consumption was significantly higher on days 8–10 than on days 1–3, in terms of both the intake volume (Student's t-test: *p* = 0.0499) and g/kg (Student's t-test: *p* = 0.0241) (Fig 1C and 1D). Using RM two-way ANOVA, we observed a significant interaction between the EtOH and the water groups (F_(9,315)_ = 2.071; *p* = 0.0371), with a significant difference in liquid intake on day 9 and 10 (Bonferroni post hoc test s: *p* < 0.05) (S1A Fig). All animals drank less on days 4 and 11 (days of saline and/or MDMA injections). We only detected significant changes in volume (RM one-way ANOVA: F_(9,99)_ = 3.232; *p* = 0.0018) (S1A Fig) and g/kg (F_(9,99)_ = 4.583; *p* < 0.0001) over time in the EtOH group, with a significant difference between day 1 and day 10, and between days 3, 9 and 10 and day 11 (Bonferroni post hoc test: *p* < 0.05, *p* < 0.01) (S1C Fig).

We observed a general though slight increase in body temperature after MDMA consumption, comparing rectal probe measurements before the first and second MDMA measurements (2 h interval) (data not shown).

### Neuronal progenitor cell survival and neuroinflammation following drug consumption

**Fig 2:**
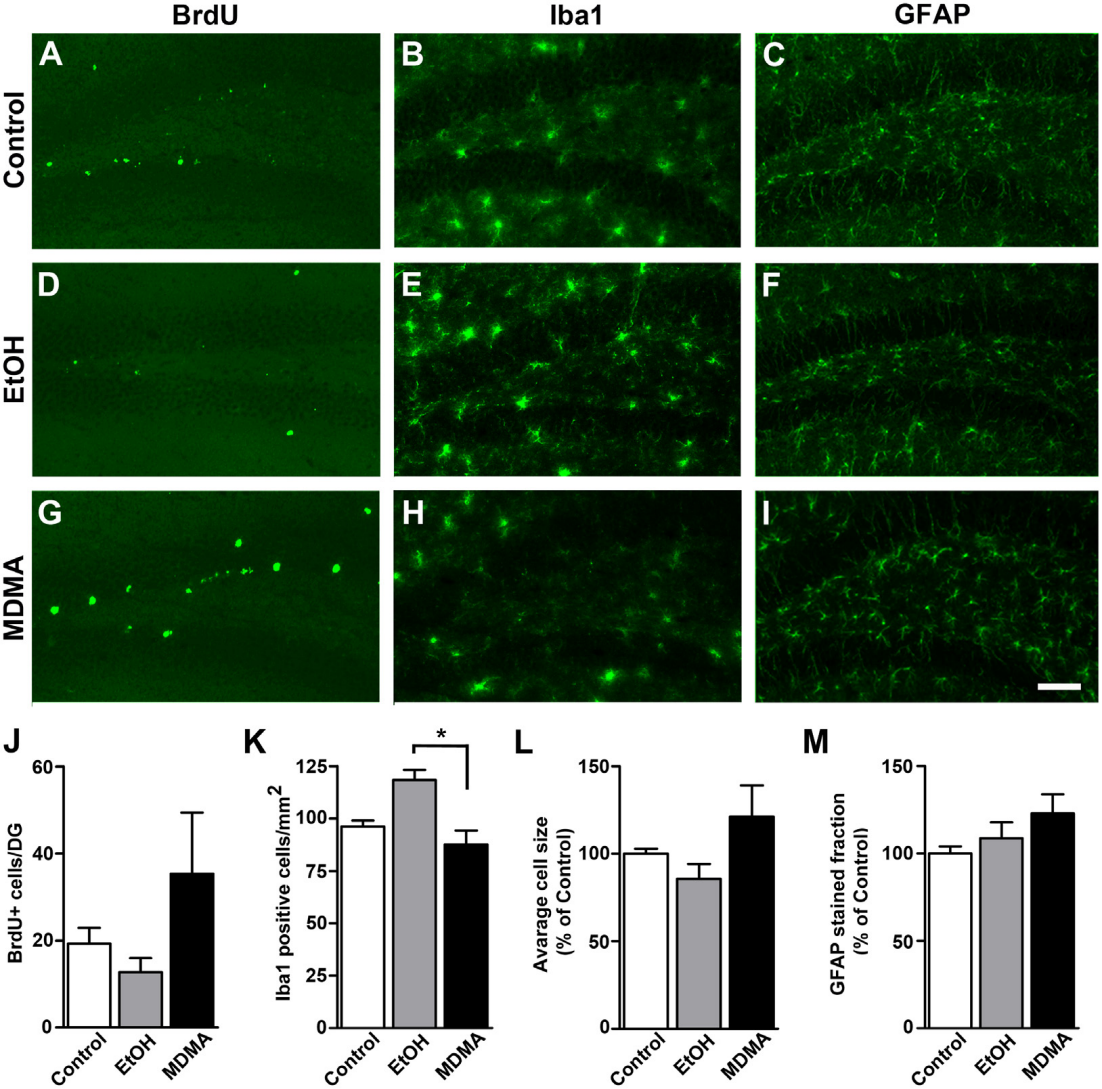
Effect of voluntary EtOH intake and high-dose MDMA (20 mg/kg x 2) on neuronal progenitor cells survival and glial cell activation in the hippocampus. Representative images show (**A,D,G**) BrdU-labeled, (**B,E,H**) Iba1-labeled and (**C,F,I**) GFAP labeled cells in the granule cell layer of the dentate gyrus. (**J**) Quantitative analysis of BrdU-labeled cells. Quantitative analysis of (**K,L**) Iba1-positive and (**M**) GFAP-positive cells from the dorsal and ventral hippocampus. Images were taken from coronal sections of the hippocampus of animals sacrificed 4 days after drug consumption. Data shown are the mean ± SEM (n = 4 mice/group). Statistically significant differences are represented by, *p < 0.0347; Tukey’s post hoc test. BrdU, Bromo-deoxyuridine; GFAP, glial fibrillary acidic protein; Iba-1, ionized calcium binding adaptor molecule 1; MDMA, 3,4-Methylenedioxymethamphetamine. Scale bar = 50μm.

Both dorsal and ventral hippocampi were analyzed 4 days after drug consumption to evaluate the survival of dividing cells and neuroinflammatory reactions. By injecting BrdU before initiating the drinking protocol and counting positive cells at the end of drug administration, we observed that neither EtOH consumption nor MDMA administration altered the number of dividing cells that survived (Fig 2A, 2D, 2G and 2J).

We did not observe microglia activation in any of the drug groups compared to controls, although we did detect a significantly increased number of Iba1+ cells/mm^2^ in animals that consumed EtOH compared to those that consumed MDMA (Fig 2E, 2H and 2K); there was no difference between the dorsal and ventral hippocampus. We observed no significant alteration in GFAP staining between groups or between the control and drug consuming groups (Fig 2C, 2F, 2I and 2M). These results suggest that there is no glia activation 4 days after drug consumption.

#### IL-1β and BDNF gene expression following drug consumption

**Fig 3:**
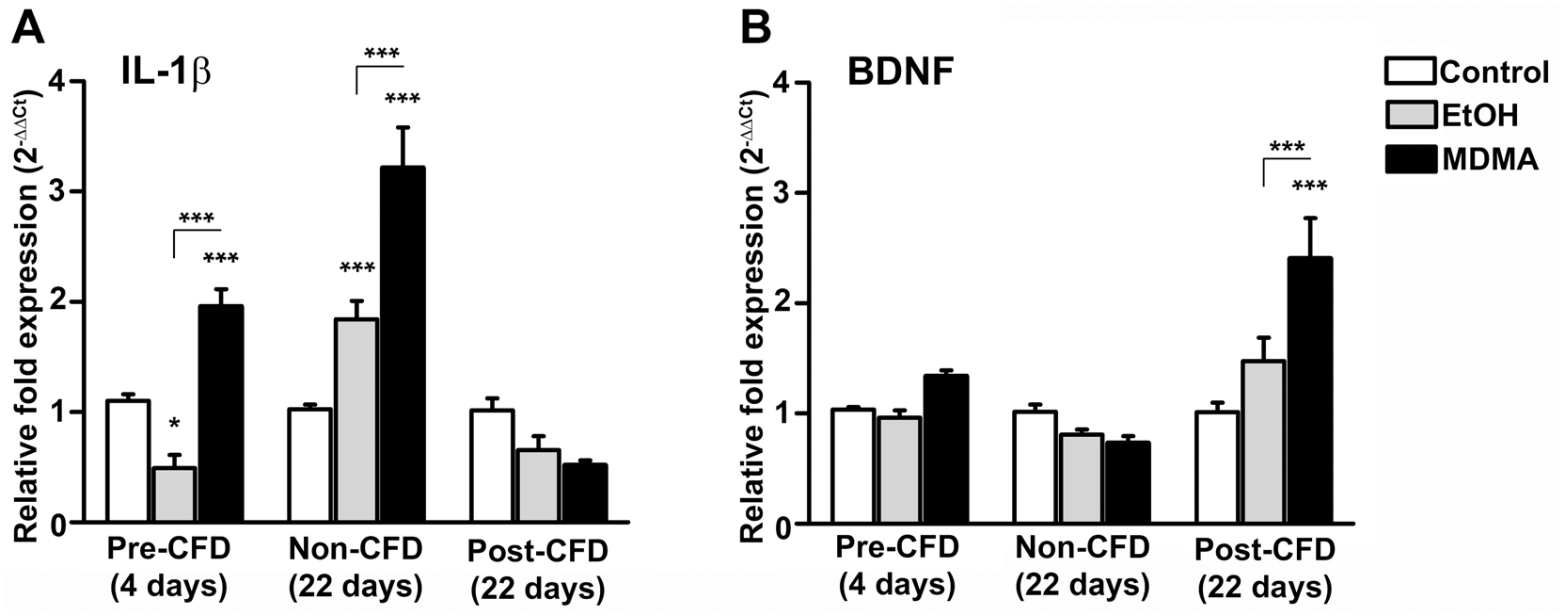
Altered IL-1β and BDNF mRNA expression following drug consumption and contextual fear discrimination. Samples were collected from the hippocampus of sacrificed animals: pre-CFD (4 days after drug consumption and 12h after behavioral studies), non-CFD (22 days after drug consumption) and post-CFD (4h after completing the CFD task and 22 days after drug consumption). Relative (**A**) IL-1β and (**B**) BDNF mRNA expression following drug consumption reveal alterations related both to drug and behavioral testing. Data shown are the mean ± SEM (n = 4–8 mice/group). Statistically significant differences between groups are represented by * (*p* < 0.05) and *** (*p* < 0.001; Bonferroni’s post hoc test). BDNF, brain-derived neurotrophic factor; IL-1β, interleukin-1beta; MDMA, 3,4-Methylenedioxymethamphetamine.

Tissue extractions from the hippocampus were assessed for alterations in gene expression at two different time points following drug consumption, first at 4 days (pre-CFD) and second at 22 days (non-CFD) the effect of drug consumption was evaluated and then, at 22 days the expression was also assessed after the contextual discrimination (post-CFD). We detected a significant reduction (~50%) in IL-1β expression in the EtOH group 4 days after drug administration, but a significant increase in the MDMA group, two-way ANOVA: F_(4,45)_ = 22.94 followed by Bonferroni post hoc test (*p* < 0,05, *p* < 0.001) (Fig 3A). BDNF expression was unaltered at the same time point, two-way ANOVA: F_(4,45)_ = 7.651 followed by Bonferroni post hoc test (Fig 3B). While the expression of IL-1β and BDNF were unaltered in the EtOH post-CFD group (22 days), MDMA administered animals showed a clear increase in BDNF expression (*p* < 0,001) and a tendency to decrease in IL-1β expression (Fig 3A and 3B). The non-CFD groups showed long-term increased IL-1β expression as a consequence of both EtOH and MDMA administration, whereas BDNF expression was unaltered in these groups (Fig 3A and 3B). In summary, these results show first that both drugs affect IL-1β expression, and second that the CFD-task affects BDNF expression in animals pre-exposed to MDMA. In addition, the CFD-task suppressed the increase in IL-1β expression observed 22 days after consumption of either drug.

### Behavioral response following drug consumption

Animals were subjected to behavioral tests to evaluate alterations in emotional responses 72h after EtOH consumption or MDMA administration. Neither anxiety-like nor depressive-like behaviors were affected by the two drugs. In addition no effect was observed on the global activity by the two drugs, which excludes any bias in the behavioral tests caused by changes in basal activity as a consequence of drug consumption (S1 Table).

#### Contextual fine discrimination following drug consumption

**Fig 4:**
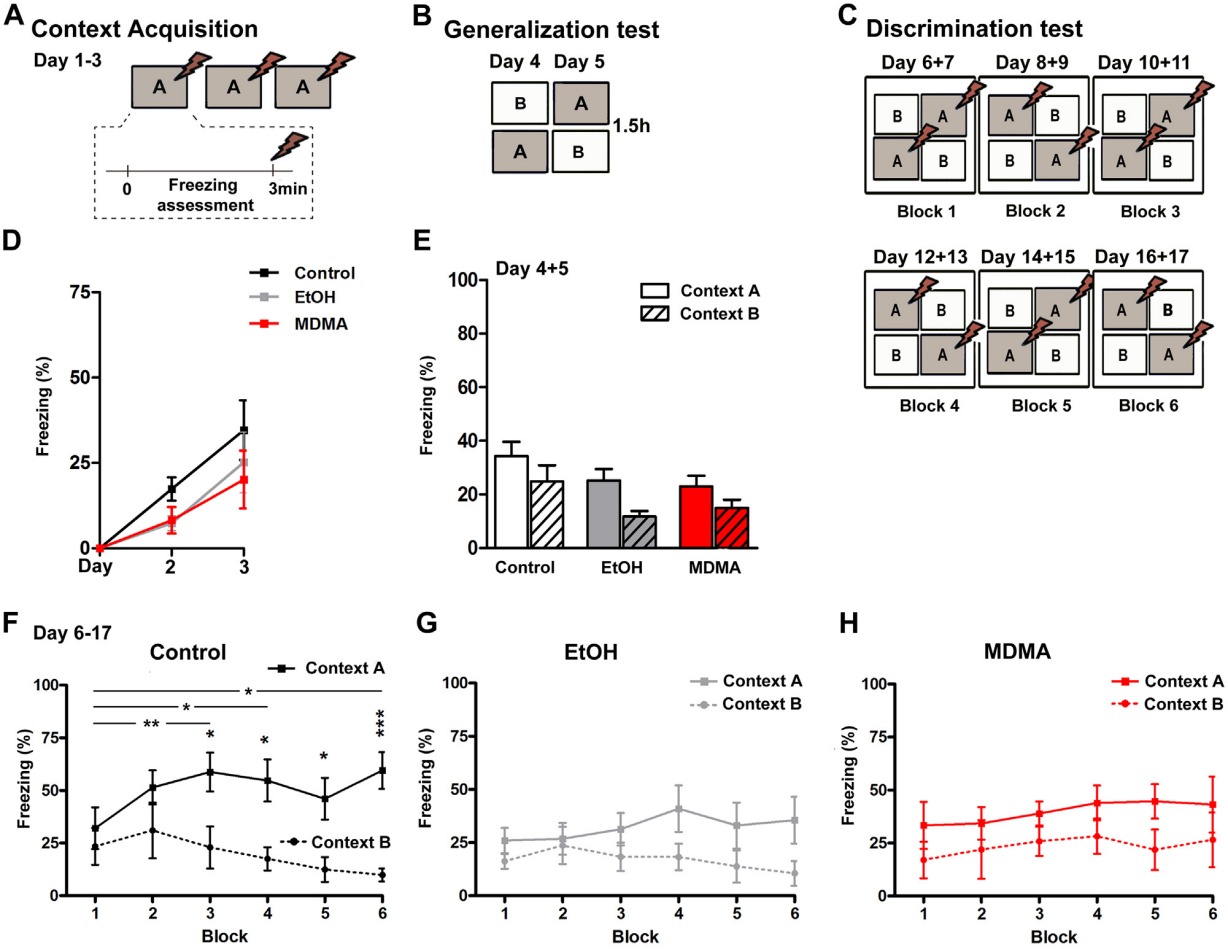
Influence of voluntary EtOH intake and high-dose MDMA (20 mg/kg x 2) on contextual fine discrimination. (**A, D**) Learning acquisition during days 1–3 was measured as freezing during 3 minutes before shock in context A. (**B, E**) Generalization, demonstrated as no significant difference in freezing between contexts A (no fill) and B (stripes), is shown for the Control (white bars), EtOH (grey bars) and MDMA (red bars) groups. (**C**) The discrimination test followed a double alternation schedule from day 6–17 and, to show graphic overviews, each 2 days were averaged together to make six blocks. (**F**) While control animals discriminate between the two contexts by day 10 (observed in block 3) of the discrimination task, both the (**G**) EtOH and (**H**) MDMA groups had impaired discrimination capacity, shown as no significant difference in the percentage of freezing time between contexts A (shock) and B (safe context). Data shown are the mean ± SEM (n = 6 mice/group). Statistically significant differences over time within each group and context are indicated by brackets and * (*p* < 0.05) and ** (*p* < 0. 01), and between context A and context B by * (*p* < 0.05), *** (*p* < 0.001; Bonferroni’s post hoc test). MDMA, 3,4-Methylenedioxymethamphetamine.

Six mice per group were tested for contextual fine discrimination (Fig 4). On days 1 to 3, we measured contextual acquisition as freezing across 3 minutes in context A pre-shock (Fig 4A); we observed no difference between groups in the percentage of freezing time on day 2 nor on day 3, evaluated by one-way ANOVA followed by Bonferroni’s post hoc test (Fig 4D). We then evaluated generalization of contexts on day 4 and 5 (Fig 4B). This part of the test revealed similar freezing time in both contexts in all groups (two-way ANOVA: F_(2,66)_ = 0.22, *p* = 0.8056), which confirms contextual generalization; again, we observed no difference between groups in context A or context B (Fig 4E) (one-way ANOVA: F_(2,33)_ = 1.709, *p* = 0.1966 and F_(2,33)_ = 2.828, *p* = 0.0735, respectively). Using the paired t-test within groups, we found a difference in freezing between the two contexts in the EtOH-group (*p* = 0.0092); however, as observed in block 1+2 (Fig 4G), generalization can be assigned to this group at a later stage of the test.

On day 6–17 the discrimination test was conducted following a double alternation schedule, with each 2 days averaged together to form six blocks (Fig 4C). Freezing levels from each day (S2 Fig) were used to calculate a block-based percentage of freezing in each context (Fig 4F–4H). While control mice showed a rapid increase in the percentage freezing time in context A (shock), reaching 59% in block 3 (Fig 4F), neither of the two drugs groups reached these levels of freezing (Fig 4G and 4H) (maximum percentage freezing time in the EtOH and MDMA groups was registered in blocks 4 (41%) and 5 (45%), respectively). The effects observed in context B (no shock) for the drug consuming groups were somewhat different: while the EtOH consuming mice showed a similar decrease in freezing time in the safe context B (Fig 4G) as that in the control group (in both the EtOH and control groups, minimum freezing (10%) was detected in block 6), MDMA-injected mice showed a slight increase in the percentage freezing time in context B throughout the test (block 1, 17%; block 6, 27%) (Fig 4H). We detected a significant increase in freezing over time in context A in the control group only (one-way ANOVA (F_(5,25)_ = 4.090, *p* = 0.0036). with a significant difference between block 1 and blocks 3, 4 and 6 (Bonferroni’s post hoc test). Similarly, we observed a significant decrease in freezing time in context B in the control group only (F_(5,25)_ = 2.979, *p* = 0.0304). Furthermore, we only observed significant differences in freezing levels between the two contexts in the drug-free group in blocks 3 to 6 (RM two-way ANOVA followed by Bonferroni’s post hoc test; *p* < 0.05, *p* < 0.001) (Fig 4F). Together, these results show that animals in both drug groups displayed problems to either discriminate between two similar contexts or associate the context to the shock. This was observed as generally low freezing levels in context A in both drug groups, and as constant or even increased percentages of freezing levels for the MDMA group in the safe context B.

## Discussion

This work underlines the influence of two common recreational drugs, EtOH and MDMA, on performance in a contextual fear discrimination task, a feature related to hippocampal activity, which may be related to impairments observed in patients with anxiety-related disorders [25].

### Drug consumption

Neuroinflammation, neuronal damage and decreased neurogenesis have been reported in rodent models of binge drinking, and following chronic EtOH administration [2,4,40]. In this study, we did not observe any clear glia activation by conventional immune staining, which may be explained by low drinking levels and the fact that 4 days had passed since the end of EtOH consumption, leaving enough time to eradicate any direct effects of ethanol on the measurements. Importantly, we did not expect that intoxication produced during the DID protocol [30] would influence behavioral or biochemical results at this point. Note that drinking behavior on days 4 and 11 in this study was influenced by the manipulation procedure (saline and/or MDMA injections), and also by acute hyperlocomotion induced by the high dose of MDMA. Thus, under our experimental conditions, the DID test produced intermittent voluntary alcohol intake, rather than binge alcohol consumption. Compared to other studies using the same protocol [30,41], we registered relatively low doses of EtOH consumption, which is likely related to the mice strain used (Swiss ICR) [42] and the manipulation schedule (see Methods). Nevertheless, EtOH affected behavior in this study and in previous ones [31], suggesting that even a low dose of EtOH has an impact on brain functions. In addition, we observed altered expression of the pro-inflammatory cytokine IL-1β in the hippocampus both 4 and 22 days after EtOH consumption, which suggests long-term agitation of the immune system as a result of intermittent alcohol intake during 2 weeks. This is in agreement with previous studies showing long term activation of microglia following EtOH intake [43].

An EtOH dose of 0.5 g/kg administered on three consecutive days is sufficient to affect cell proliferation (for review see [44]). However, even if the animals in our study reached ~1.8 g/kg alcohol intake by the end of the test, we did not detect alterations in neuronal progenitor cells survival. This is consistent with observations in mice following voluntary EtOH drinking during 28 days [4]. Regarding the effects of MDMA, and given that the MDMA dose used produces a neuroinflammatory reaction [29,31,45], the slight increase in BrdU+ cells that we observed in this group could indicate neuronal damage and consequent cell cycle re-entry [46]. The amount of Iba1-positive cells was significantly higher in EtOH consuming compared to MDMA administered mice 4 days after drug consumption. This could reflect dissimilarity in the time course of microglial response in the hippocampus seeing that EtOH induce morphological changes to microglia that can be appreciated even 28 days after consumption [43] while MDMA transiently activates microglia at early time points (before 24h) [47]. One could speculate that the small increase of Iba1-positive cells might reflect recruition of microglia to the hippocampal area while the equivalent cell-size of microglia between groups could denote the absence of activation. This is in accordance with the non-detected increase in cytokine expression (at 4 days) that normally occurs when microglia is activated [48]. It is noteworthy that partial but prolonged microglia activation is reported after EtOH consumption [43,48].

### IL-1β expression

We observed decreased IL-1β expression 4 days after alcohol consumption, and also following the CFD task at 22 days after alcohol intake (post-CFD), compared to the increased detected in the non-CFD group at 22 days. While a complete understanding of these results requires additional experiments that are beyond the scope of this work, there are several plausible explanations. For instance, the observed decrease in IL-1β expression post-CFD may be related to anti-inflammatory effects caused by suppression of pro-inflammatory gene networks, given that both EtOH and stress (such as a foot-shock) activate the hypothalamic-pituitary-adrenal (HPA) axis, leading to glucocorticoid release [49–51]. Furthermore, pre-exposure to stressful situations as a result of the behavioral tests, which were conducted 12h before sample collection on the 4^th^ day, could also activate a sensitized (by cytokines) HPA-axis [52]. Alternatively, the decreased IL-1β expression could reflect immune-to-brain communication [53], given that a single dose of EtOH has been shown to induce a significant reduction in peripheral cytokine levels [54]. The same authors also show a correlation between liver, blood and brain cytokine levels following alcohol administration [54]. In contrast, several reports demonstrate that chronic EtOH administration is related to enhanced cytokine production in the periphery and the brain (e.g. [55,56]), which is consistent with our observations in the non-CFD group at 22 days after EtOH consumption. Finally, alcohol withdrawal is also known to increase cytokine production [57] and enhance the HPA-axis response [58], which further corroborates our results in both the non-CFD and post-CFD groups at day 22, respectively. Taken together, our results indicate a relationship between EtOH and IL-1β expression, consistent with previous reports [18,54–56].

In accordance with previous studies in rats [59], we observed an increase in IL-1β expression in the hippocampus at 4 and 22 days after MDMA administration. Regarding the effects of stress on the HPA axis mentioned above, acute MDMA administration has been found to induce glucocorticoid secretion without affecting proinflammatory cytokines [60]. In contrast, the decrease in IL-1β expression observed at day 22 (comparing post-CFD and non-CFD) could reflect MDMA-induced immunosuppression by priming the HPA-axis, as reported by Connor *et al*. [60]. Additional studies on glucocorticoid levels at both time points would help to further elucidate the relationship between this pathway and both EtOH and MDMA. Nevertheless, our results show that the long term increase in IL-1β expression following drug consumption is reversed by the CFD test, probably by priming the HPA-axis as previously suggested [52]. Furthermore, our study reflects the difficulties in relating stress, alcohol and neuroinflammation, as addressed by Deak *et al*. [61], since these processes are modulated by many factors like timing, dose and duration.

### BDNF expression

Neither drug appeared to affect BDNF expression, which is consistent with previous reports on the effect of alcohol on BDNF [62], but in disagreement with a decrease expression in the hippocampus following MDMA administration reported by Martinez-Turrillas *et al*. [63]. Interestingly, the same authors also showed increased phosphorylation of cAMP response element-binding protein (CREB) in the same brain region, which is intriguing in light of the fact that BDNF is known to have a CRE-site in its promoter [64]. This, together with the CRE-related induction of BDNF expression after fear conditioning [65], may partly explain the increased BDNF expression observed in the post-CFD MDMA group compared to the EtOH and control animals. Our results are consistent with published work showing an increase in BDNF expression after MDMA exposure as a consequence of training in a behavioral test [66]. Furthermore, (as discussed above) MDMA has immunosuppressive effects and enhances the sensitivity of the HPA-axis to subsequent stressors [60,67,68], which may be another plausible aspect potentiating the stress-induced increase in BDNF as previously described by Revest *et al*. [69]. Our data do not allow us to determine whether the CFD-protocol *per se* influence basal IL-1β or BDNF expression, although such relationship is confirmed in previous reports [65,70,71].

Interestingly, BDNF is involved in modulating the synthesis and phosphorylation of Synapsin-1, a key phosphoprotein important for fine tuning synapses by affecting neurotransmitter release, among others important for stress-related memory formation [72]. We previously demonstrated oxidative modulation of Synapsin-1 in the hippocampus following consumption of both EtOH and MDMA [29]. Taken together, one may speculate that BDNF compensates for ineffective Synapsin-1 and the consequent disruption of neurotransmitter release. However, BDNF-mediated neurotransmission is strongly attenuated by Synapsin deletion [73]. Furthermore, there is a notable absence of correlation between BDNF expression and discrimination ratio post-CFD (S3D Fig). Consequently, oxidative modulation of Synapsin-1 may underlie the perturbations we observed in the discrimination task. Nevertheless, both IL-1β and BDNF are recognized as key players (upstream of Synapsin-1) in synaptic plasticity [19–21], and their dysregulation may also correspond to hippocampal related memory impairments (for review see [74]).

### Drugs and behavior

Ours results, along with previous findings [31] reveal that neither of these two drugs, given alone, affects anxiety or depressive-like behaviors. Pascual *et al*. [2] reported that rodents have impaired hippocampal-dependent learning in the object recognition test 24h after EtOH administration. However, consistent with the contextual learning that we observed here during days 1–3, this effect is reversed 72h after EtOH exposure. In contrast, the same test (novel object recognition) revealed memory deficits 72h after MDMA administration [29], leaving our results difficult to relate to this specific contextual learning task. Note that MDMA also has been shown to improve spatial learning in the water maze, which corresponds to a training-induced rise in the number of spines, and in BDNF expression [66]. We observed altered performance in the CFD task after drug consumption, which raises the possibility that increased spine density also could have occurred in our animals, and that the fine tuning acquired in the hippocampus during the learning process was hampered by insufficient pruning of “unused” synapses. In general, we speculate that impaired performance in the CFD-task may be related to cognitive features that require a well-adjusted hippocampus, such as associative learning or the capacity to distinguish one context from another [34,75].

Notably, the generalization between contexts observed in MDMA-administered animals differed from that observed in the EtOH group. While the MDMA animals generalized between the two contexts throughout the test (no change in freezing levels in either context), the EtOH group showed decreased freezing in the safe context, and maintained low freezing levels in the shock-context. Consistent with our results, low freezing is reported to be a consequence of ethanol injection before contextual fear conditioning [76]. In addition, while our results indicate a negative correlation between freezing behavior and IL-β expression in the hippocampus of alcohol-consuming animals (S3E Fig), no such relation was detected in the MDMA group (S3G Fig).

## Conclusions

In this study, we have shown impaired performance in a contextual fear discrimination task following EtOH consumption or a high dose of MDMA. Both drugs affected immune-activated pathways which resulted in alterations of the proinflammatory cytokine IL-1β and of BDNF mRNA expression and IL-1β expression was even affected 22 days after intermittent EtOH consumption. Furthermore, animals pre-exposed to the drugs appeared to be more sensitive to stressors. Although the animals from the two drug groups performed somewhat differently, we postulate that the observed deficiency in both cases could reflect insufficient adaptation of hippocampal synaptic plasticity caused by different molecular mechanisms that was detected as generalization in the CFD task in both drug groups. Additional studies are needed to fully understand the consequence of drug consumption on vulnerability to anxiety-related disorders, and the interplay between inflammatory markers and external stressors in psychiatric disorders.

A detailed understanding of the molecular mechanisms that mediate cognitive impairment in psychiatric disorders will provide us with novel and more selective targets for drug development, and help to improve diagnosis in these patients.

## Supporting Information

S1 Fig Drinking behavior. Volume (ml) of water or EtOH intake as registered each day during (**A**) first and (**B**) second round of experiments. (**C**) EtOH consumption calculated as g/kg from first round of experiments (shown with a line) and from second round of experiments (dotted line). Statistically significant differences (first round of experiments) in (**A**) volume of water and EtOH intake and for (**C**) EtOH (g/kg) consumption between day 1 and day 10 and between days 3,9,10 and day11 are indicated by brackets and ** (*p* < 0.01) and * (*p* < 0.05; Bonferroni’s post hoc test). Mean ± SEM of all animals are shown (n = 12–24 mice/group). MDMA, 3,4-Methylenedioxymethamphetamine.
(TIF)

S2 Fig Influence of voluntary EtOH intake and high-dose MDMA (20 mg/kg x 2) administration on contextual fine discrimination. The discrimination test followed a double alternation schedule on days 6–17. (**A**) Control animals learned to discriminate between the two contexts by day 10 of the discrimination task, while both the (**B**) EtOH-consuming and (**C**) MDMA administered animals never learned to separate the safe and unsafe contexts, reflected by the absence of a significant difference in the percentage of freezing time between contexts A (shock) and B (safe context). Data shown are the mean ± SEM (n = 6 mice/group). Statistically significant differences between context A and context B are represented by * (*p* < 0.05; Bonferroni’s post hoc test). MDMA, 3,4-Methylenedioxymethamphetamine.
(TIF)

S3 Fig Influence of voluntary EtOH intake and high-dose of MDMA (20 mg/kg x 2) administration on inflammatory markers and the CFD-taks. Correlation analysis between mean EtOH intake during day 8–10 and IL-1β and BDNF mRNA expression (**A**) 4 and (**B**) 22 days after the DID test. (**C**) Relationship between EtOH consumption and mean freezing levels (%) from day 6–17 in the CFD task or discrimination ratio from block 6 (day 16–17). (**D**) Relationship between discrimination ration and IL-1β or BDNF mRNA expression. (**E**) Correlation between mean freezing time (%) from day 6–17, EtOH intake and IL-1β expression or (**F**) BDNF expression. (**G**) Correlation between mean freezing and IL-1β or BDNF expression and (**H**) discrimination ratio and cytokine expression after MDMA administration. Each individual animal is represented by a dot, square or a triangle, and significant correlations assessed by Pearson analysis are indicated by a line. BDNF, brain-derived neurotrophic factor; CFD, contextual fear discrimination; IL-1β, interleukin-1beta; MDMA, 3,4-Methylenedioxymethamphetamine.
(TIF)

S1 Table Locomotor Activity, Anxiety-, and Depressive-like Behavior 72h after alcohol consumption and MDMA administration. (TIF)
