# Supplementary figures and images for: Reduced Contextual Discrimination following Alcohol Consumption or MDMA Administration in Mice
Source: PLoS One. 2015 Nov 13;10(11):e0142978. doi: 10.1371/journal.pone.0142978 (PMC4643963; doi:10.1371/journal.pone.0142978)

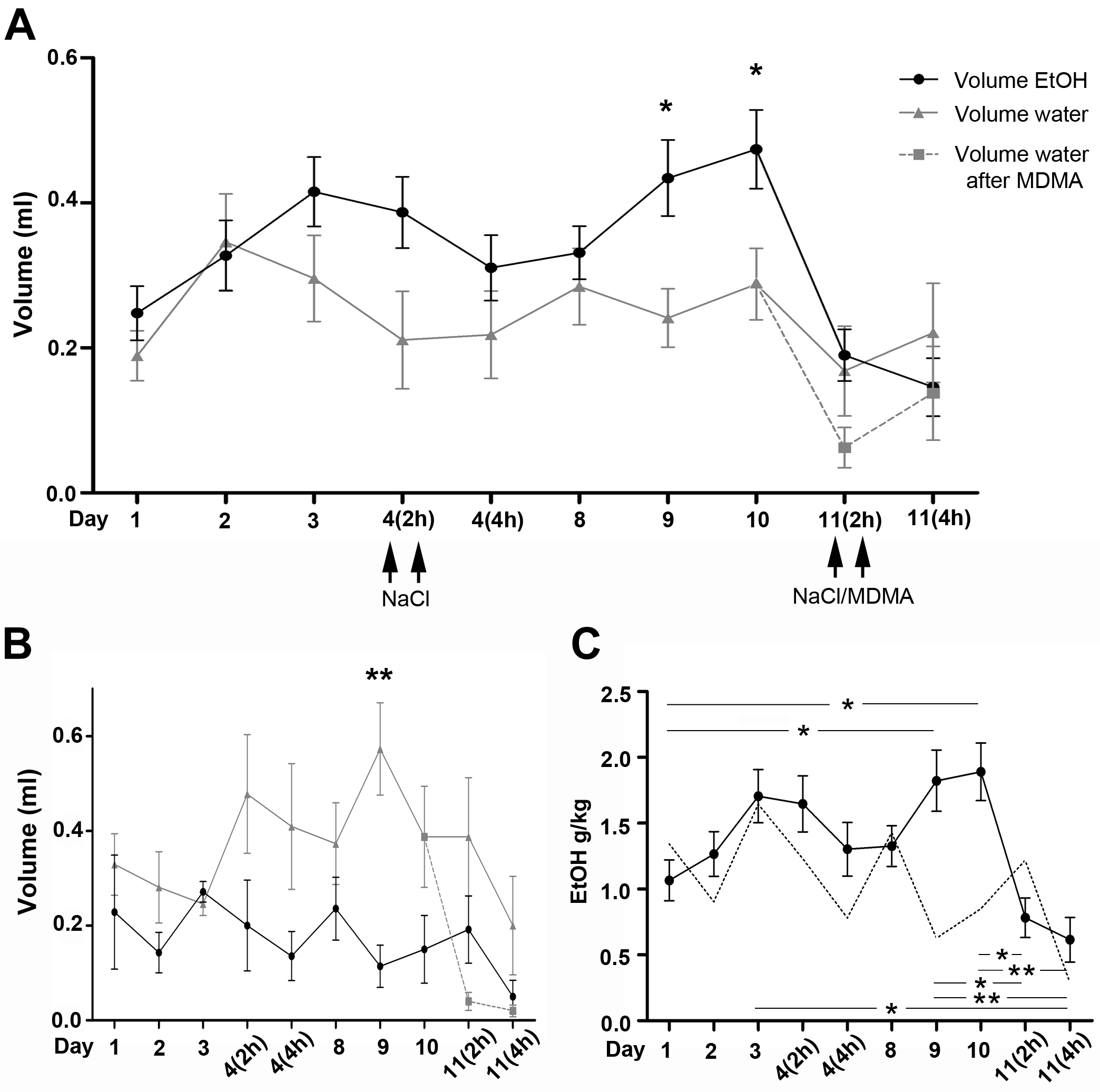

Supplement: S1 Fig — Volume (ml) of water or EtOH intake as registered each day during (A) first and (B) second round of experiments. (C) EtOH consumption calculated as g/kg from first round of experiments (shown with a line) and from second round of experiments (dotted line). Statistically significant differences (first round of experiments) in (A) volume of water and EtOH intake and for (C) EtOH (g/kg) consumption between day 1 and day 10 and between days 3,9,10 and day11 are indicated by brackets and ** (p < 0.01) and * (p < 0.05; Bonferroni’s post hoc test). Mean ± SEM of all animals are shown (n = 12–24 mice/group). MDMA, 3,4-Methylenedioxymethamphetamine. (TIF) [file pone.0142978.s001.tif]

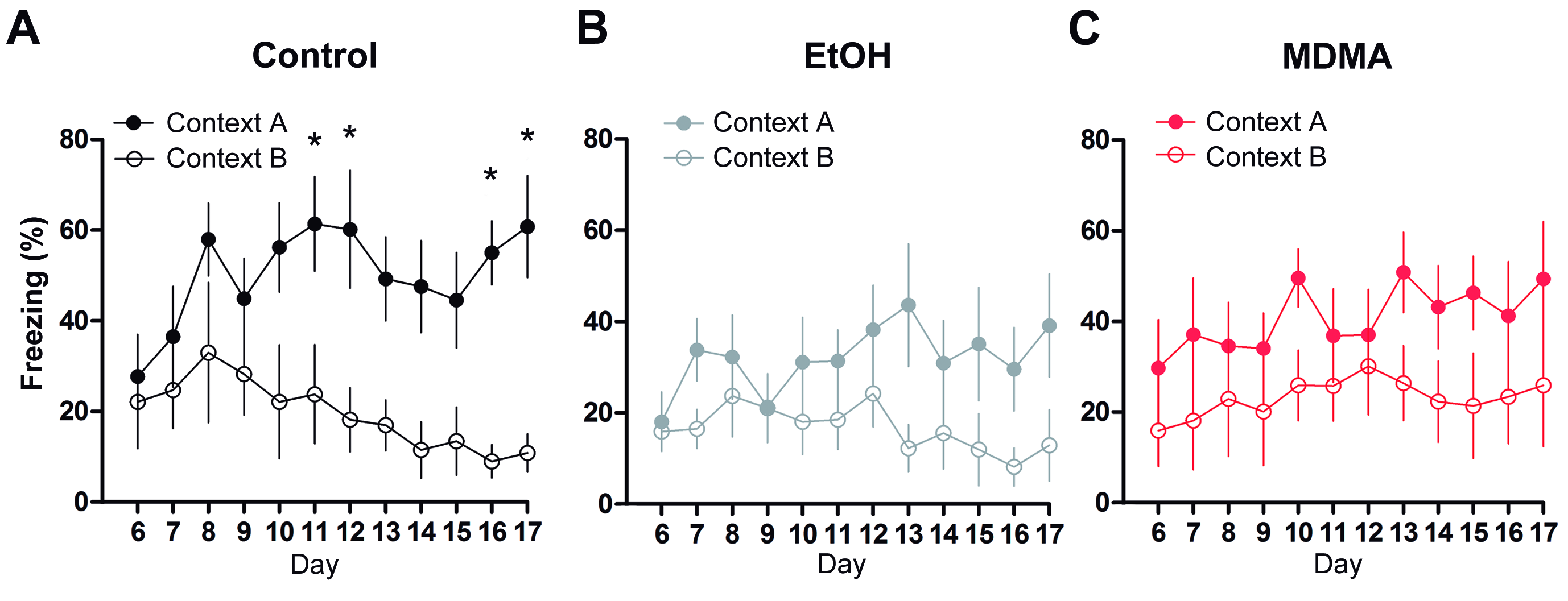

Supplement: S2 Fig — The discrimination test followed a double alternation schedule on days 6–17. (A) Control animals learned to discriminate between the two contexts by day 10 of the discrimination task, while both the (B) EtOH-consuming and (C) MDMA administered animals never learned to separate the safe and unsafe contexts, reflected by the absence of a significant difference in the percentage of freezing time between contexts A (shock) and B (safe context). Data shown are the mean ± SEM (n = 6 mice/group). Statistically significant differences between context A and context B are represented by * (p < 0.05; Bonferroni’s post hoc test). MDMA, 3,4-Methylenedioxymethamphetamine. (TIF) [file pone.0142978.s002.tif]

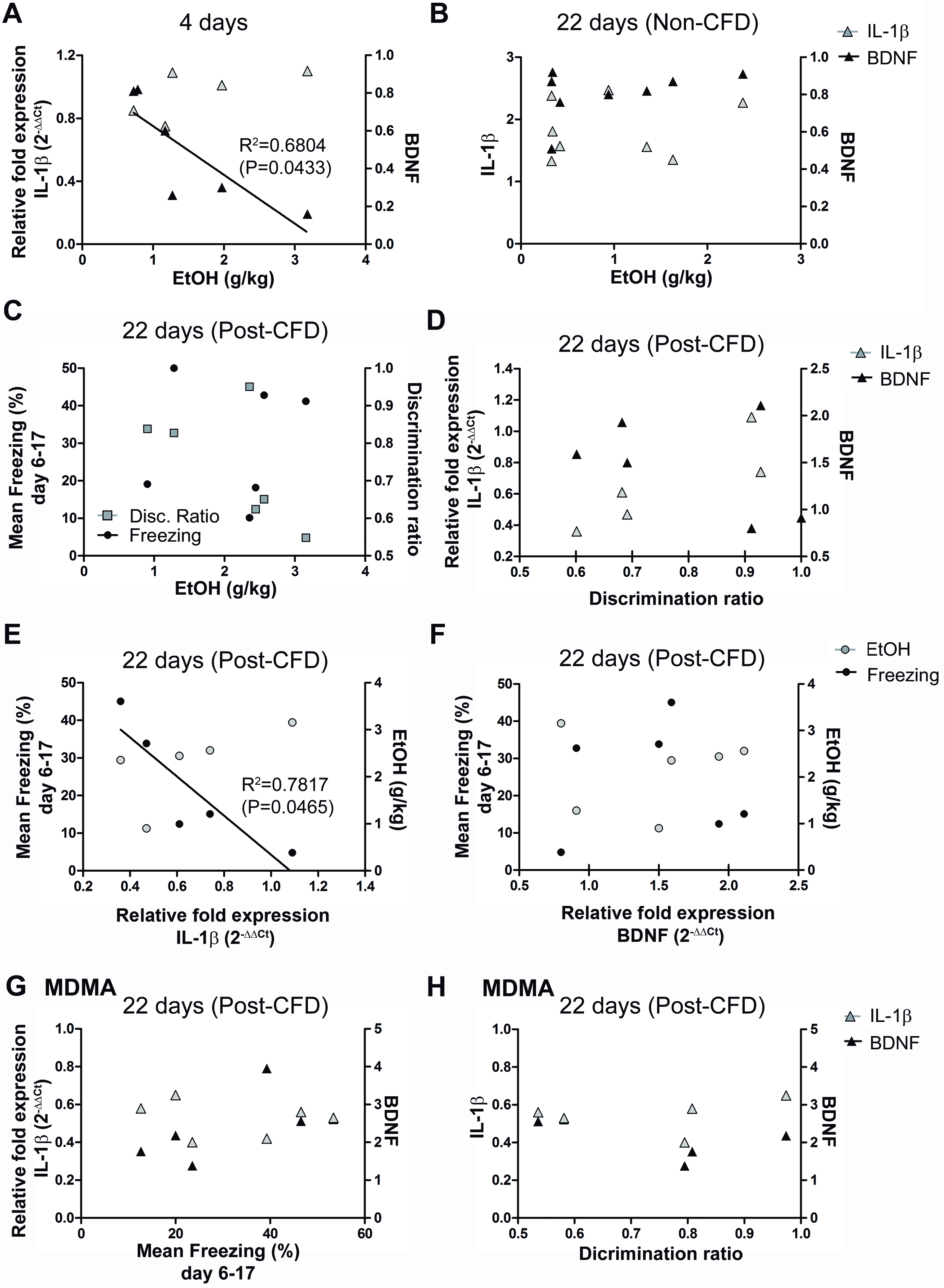

Supplement: S3 Fig — Correlation analysis between mean EtOH intake during day 8–10 and IL-1β and BDNF mRNA expression (A) 4 and (B) 22 days after the DID test. (C) Relationship between EtOH consumption and mean freezing levels (%) from day 6–17 in the CFD task or discrimination ratio from block 6 (day 16–17). (D) Relationship between discrimination ration and IL-1β or BDNF mRNA expression. (E) Correlation between mean freezing time (%) from day 6–17, EtOH intake and IL-1β expression or (F) BDNF expression. (G) Correlation between mean freezing and IL-1β or BDNF expression and (H) discrimination ratio and cytokine expression after MDMA administration. Each individual animal is represented by a dot, square or a triangle, and significant correlations assessed by Pearson analysis are indicated by a line. BDNF, brain-derived neurotrophic factor; CFD, contextual fear discrimination; IL-1β, interleukin-1beta; MDMA, 3,4-Methylenedioxymethamphetamine. (TIF) [file pone.0142978.s003.tif]

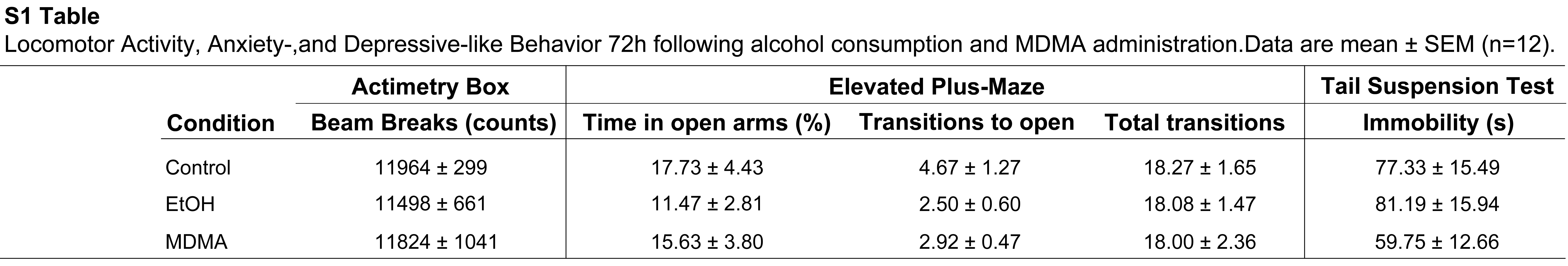

Supplement: S1 Table — (TIF) [file pone.0142978.s004.tif]
